# A basilosaurid archaeocete (Cetacea, Pelagiceti) from the Late Eocene of Oregon, USA

**DOI:** 10.7717/peerj.9809

**Published:** 2020-10-02

**Authors:** Mark D. Uhen, David Taylor

**Affiliations:** 1 Department of Atmospheric, Oceanic, and Earth Sciences, George Mason University, Fairfax, VA, USA; 2 Department of Geology, Portland State University, Portland, OR, USA

**Keywords:** Cetacea, Biogeography, Eocene, Pacific Ocean

## Abstract

**Background:**

Basilosaurid archaeocetes are known from the Late Eocene of virtually all coastlines bearing coeval marine rocks except the North Pacific Basin, until now. Here we report on three consecutive posterior thoracic vertebrae of a large, basilosaurid archaeocete from a Late Eocene horizon in the Keasey Formation in Oregon.

**Methods:**

These vertebrae were morphologically and morphometrically compared to other vertebrae of similar age from around the world.

**Results:**

The specimens were determined to be different from all currently named species of fossil cetacean, but most similar to those found in the Gulf Coast region of North America. These vertebrae represent the first confirmed specimen of a Late Eocene basilosaurid from the North Pacific. These and other basilosaurids known only from vertebrae are reviewed here in the context of Late Eocene paleoceanography and cetacean evolution.

## Introduction

**Figure 1:**
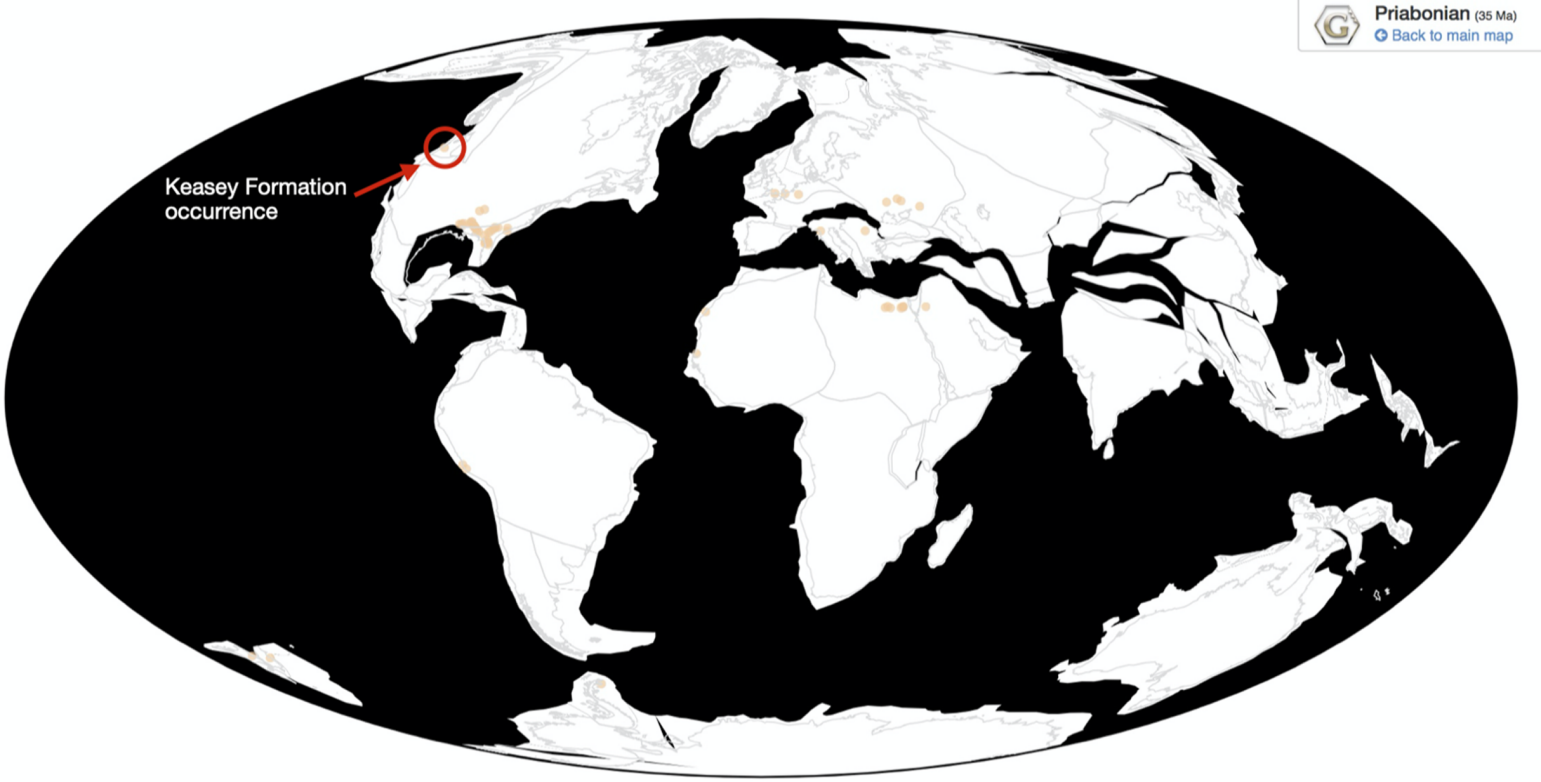
Map of Eocene basilosaurids. All published occurrences of basilosaurid archaeocetes from the Priabonian (Late Eocene). The Keasey Formation occurrences are circled in red. Data and map are from the Paleobiology Database (paleobiodb.org). Occurrences are from the references therein and listed in Supplemental References.

Cetaceans originate during the late Early Eocene (Ypresian) in the Indo-Pakistan region (Bajpai & Gingerich, 1998). From there, semi-aquatic cetaceans (mainly Protocetidae) disperse around the world rapidly, being firmly established in Egypt (Bebej et al., 2015), West Africa (Gingerich & Cappetta, 2014), Eastern North America (Hulbert et al., 1998), and even Peru (Lambert et al., 2019) during the early Middle Eocene (Lutetian). By the late Eocene (Priabonian), the fully aquatic basilosaurid archaeocetes are known from almost all continents (see Fig. 1; except Australia, although they are known from New Zealand). Collecting localities that have produced basilosaurids are concentrated around the closing Tethys Sea, the western North Atlantic, and West Africa. These areas represent the northern tropics to subtropics in the late Eocene (see Fig. 1). In addition to these regions, basilosaurids are also known from West Africa, New Zealand, Peru, and Seymour Island, Antarctica. These additional localities indicate that while finds are concentrated in the northern tropics and subtropics, basilosaurid archaeocetes are restricted neither to these areas nor these environments.

The cetacean vertebrae from the Keasey Formation reported on here were briefly mentioned by Goedert (1988), but it was not clear in that publication if the vertebrae were from the Late Eocene (Priabonian) or Early Oligocene (Rupelian) portion of the Keasey Formation, nor what type of cetacean the vertebrae were from. Until now, no confirmed finds of late Eocene Cetacea, including basilosaurids have been confirmed from the entire North Pacific Ocean basin. Here we report on the first such confirmed find from the Priabonian part of the Keasey Formation, western Oregon. We also report on other large basilosaurids from other areas known only from vertebrae. These tantalizing specimens hint at additional basilosaurid diversity in the late Eocene from other areas of the world as well, and perhaps suggests sorting of these species based on environmental preferences.

## Materials and Methods

The late Ralph Keasey, former land manager for the Keasey Family Corporation, gave permission in the late 1980s for us to conduct our field work and obtain the whale specimen described below.

## Systematic Paleontology

**Cetacea Brisson 1756**

**Pelagiceti Uhen 2008**

**Basilosauridae Cope 1868**

Basilosauridae gen. et sp. indet.

**Specimen:** NWMNH 2151, three sequential posterior thoracic vertebrae.

**Collecting Locality:** PBDB Collection 206340. Rock Creek, Oregon, about five miles (eight km) west of Vernonia, near the road from Vernonia to Keasey (Keasey Road) (45.87559 N, 123.313797 W). Fossil localities in this area are all from the lower and middle members of the Keasey Formation (Hickman, 1976) and the sites are late Eocene (Refugian locally and Priabonian globally) in age (Nesbitt, 2018; also, see below). Also, see Fig. 1.

**Geologic and Stratigraphic Context:**

The Late Eocene to Early Oligocene Keasey Formation is a gray volcaniclastic siltstone—mudstone marine unit up to approximately 700 m thick and deposited in a forearc setting. It includes three informal members which in ascending stratigraphic order are a lower member up to 150 m thick, a 500 m thick middle member, and an approximately 50 m thick upper member as described in Hickman (1976).

The Keasey Formation is disconformably bounded below by the middle Eocene Cowlitz and Hamlet formations, and above by the lower Oligocene Pittsburg Bluff and Sager Creek formations (Prothero & Hankins, 2000). The lower member ranges up to 150 m in stratigraphic thickness and consists of highly tuffaceous dark gray, micaceous siltstone and mudstone. Many of the beds are laminated and numerous horizons are glauconitic. The middle member is 500 m thick and is composed of highly micaceous light gray siltstone and mudstone with occasional ash beds. There are common concretionary horizons, as well. The upper member is 50 m thick and is composed of alternating light and dark gray tuffaceous siltstone and mudstone. There are numerous well indurated calcareous beds and concretionary horizons. This member tends to be more resistant to erosion than the other two members and as a result may be a ridge-former (Hickman, 1976).

The cetacean vertebrae are from the middle member, which is composed of light-gray highly tuffaceous siltstone and mudstone. The thick mudstone units are thoroughly bioturbated, giving them a massive appearance. PBDB Collection 206340 is on Rock Creek on the beveled terrace of the streambed and is from the lower part of the middle member. Other vertebrates from the Keasey Formation are limited, but include: bony fish *Probathygadus keaseyensis* (David, 1956); sharks *Centrophorus* sp. (Welton, 1972), *Heptranchias howellii* (Welton, 1974), *Keasius taylori* (Welton, 2013), *Notorhynchus* sp. (Welton, 1972), *Odontaspis* sp. (Welton, 1972), *Oligodalatias jordani* (Welton, 2016b), *Orthechinorhinus davidae* (Welton, 2016a), *Squatina* sp. (Welton, 1972); and marine birds *Phocavis maritimus* (Goedert, 1988), aff. *Argillornis* sp. (Goedert, 1989). Many invertebrate fossils have also been described that help to characterize the environment of the Keasey Formation. Most of these fossil occurrences have been entered into the PBDB and are well illustrated by the following studies: mollusks (Hickman, 1976, 1980); crinoids (Burns, Campbell & Mooi, 2005); crabs (Rathbun, 1932), and microfossils (McDougall, 1975).

**Chronology/Biochronology:**

The age of the Keasey Formation is now reasonably well understood, although historically there had been difficulties in defining its precise age as well as the position of the Eocene/Oligocene boundary within it (Hickman, 1976, 2003; Prothero & Hankins, 2000; Nesbitt, 2018). The position of the epoch boundary within the formation is of special interest here, since basilosaurids characteristically are Late Eocene (Priabonian) in age. Paleomagnetic work (Prothero & Hankins, 2000) suggests, as discussed below, positioning the epoch boundary high in the formation. Thus, the occurrence of the basilosaurid vertebrae in the lower part of the middle member of the Keasey Formation is consistent with a Late Eocene age assignment.

McDougall (1975) described benthic foraminifera from the lower Member and lower part of Middle Member of the Keasey Formation along Rock Creek. The earliest strata in that section are toward the southwest, near the defunct town of Keasey, and the higher beds are toward the northeast. McDougall (1975; see Fig. 3) section stops about 400 m west of the whale locality. The Lower Member is referred to the Narizian Stage while the lower part of the middle member is allocated to the *Sigmomorphina schenki* Zone (based in Washington and coeval with the *Uvigerina cocoaensis* Zone of California). Sample KAM 1043 (from about 400 m west of the whale site) yields *U. cocoaensis* and *Eponides gaviotaensis*. It may be referred to the lower Refugian as it does not yield any upper Refugian species (McDougall, 1975). Thus, while strict biochronologic control is lacking, the whale locality is in proximity to Lower Refugian benthic foraminiferal faunas.

Later, paleomagnetic work (Prothero & Hankins, 2000) was conducted in the Keasey Formation along Rock Creek, coincident with the stratigraphic section of McDougall (1975). Prothero & Hankins (2000) highest paleomagnetic sample is roughly 2,700 m west of the whale locality (perhaps only about 50 m stratigraphically below of the whale site). They (Prothero & Hankins, 2000) noted that the lower member exhibits reversed polarity while the superjacent 105 m of the Middle Member along Rock Creek reveal normal polarity (allocated to C15n). Magnetic stratigraphic results from their Sunset Highway Section reveal a superjacent interval within the Keasey Formation having reversed polarity, which they allocate to C13r based on correlation with the Lincoln Creek Formation in Washington (Prothero & Armentrout, 1985; Prothero & Hankins, 2000). They (Prothero & Hankins, 2000) placed the Eocene/Oligocene boundary in the upper part of C13r, near the top of the Middle Member of the Keasey Formation (Hickman, 2014).

Provided that the magnetic reversal sequence (Prothero & Hankins, 2000) is correct, the stratigraphic position of the whale vertebrae low in the Keasey Formation, and proximity of the vertebrate locality to lower Lower Refugian benthic foraminifera, suggests a Late Eocene (Priabonian) age. Hickman (2015) also placed a methane cold seep site from the basal part of the Middle Member (UCMP IP16004; PBDB Collection 206340) in the Eocene (Priabonian). The cold seep is a few hundred meters east of the whale locality.

**Environment:** A review of molluskan faunas based on the taxonomic composition of the benthic communities in the Keasey Formation (Hickman, 1976, 2003, 2014) indicates a predominantly deep-water bathyal environment although depths as shallow as outer neritic have been suggested, as well (Hickman, 1980). This depth range is consistent with that reported for benthic foraminifera (McDougall, 1975).

There was a marked regional Late Eocene climatic deterioration that is well documented within the Pacific Northwest (Hickman, 1984, 2003). The climatic change is recognized by the shift from the Middle Eocene tropical Cowlitz benthic faunas (Nesbitt, 1995) to cool-water Late Eocene faunal lineages characteristic of the Keasey Formation (Hickman, 1976, 1980). Note that the regional transition from the “tropical” Cowlitz to the “cool-water” Keasey faunas occurs at about 36.5 Ma, which precedes the negative oxygen and positive carbon isotope anomalies dating to about 33.5 Ma and inferred to represent the terminal Eocene global cooling episode (Hickman, 2003).

**Figure 2:**
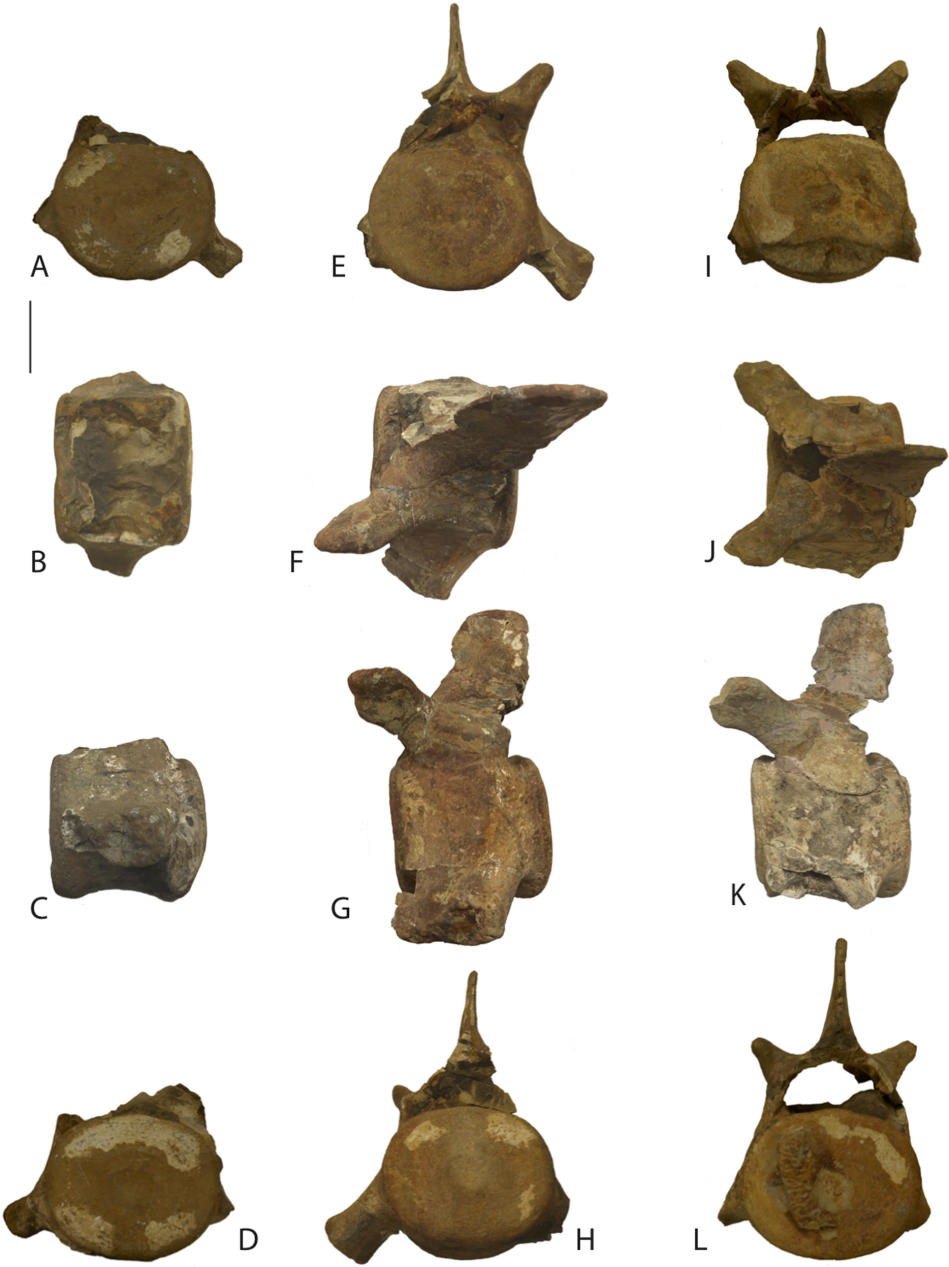
Keasey basilosaurid specimen. NWMNH 2151, three posterior thoracic vertebrae. Images in the first column (A–D) are vertebra A in anterior, dorsal, left lateral, and posterior views. Images in the second column (E–H) are vertebra B in anterior, dorsal, left lateral, and posterior views. Images in the third column (I–L) are vertebra C in anterior, dorsal, left lateral, and posterior views. Scale bar is 10 cm.

**Morphological Description:** Each of the three vertebrae are large, generally equidemensional vertebrae with intact epiphyses firmly attached to the vertebral bodies (Fig. 2). Given their unknown positions, they will be referred to as A, B, and C. Size and morphological indicators presented below suggest that they are sequential from anterior (A) to posterior (C) and comparison with other basilosaurids indicate that they represent posterior thoracic vertebrae. Measurements of the vertebrae are listed in Table 1.

**Table 1:** Vertebral measurements.

Vertebra	AW	AH	VL	PW	PH	DL	TPH
A	176	142	139	169	146	138	42*
B	171	145	136	181	150	144	46
C	178	149*	140*	185	156	148	29

**Notes:**

Vertebral measurements, in mm. Vertebral measurements are after those in Uhen (2004).

* Estimated measurement due to minor breakage.

Vertebra A (Figs. 2A–2D) is missing the right transverse process and neural arch. The right neural pedicle is mostly present and the bases of the transverse processes are present, as is the left transverse process. The positions of the bases of the transverse processes are a bit higher on the vertebral body than in B or C, suggesting it is the most anterior of the three vertebrae. In addition, the distal end of the left transverse process displays a spongy, concave surface indicative of a single articular surface for a rib. The articular surface is slightly larger than the one in vertebra B and the transverse process itself is slightly shorter, suggesting it is anterior to vertebra B in sequence.

Vertebra B (Figs. 2E–2H) is mostly intact, missing only the postzygapophyses, right prezygapophysis and most of the right transverse process. The intact left transverse process angles slightly ventrally. The neural spine and transverse processes are anteroposteriorally broad. The left prezygapophysis has a generally bulbuous appearance and lacks well defined articular surfaces. The left transverse process displays a spongy, concave surface indicative of a single articular surface for a rib. This is best interpreted as a posterior thoracic vertebra, near the end of the thoracic series.

Vertebra C (Figs. 2I–2L) is similarly preserved to vertebra B: missing the postzygapophyses, right prezygapophysis and most of the right transverse process, except the left transverse process is broken off and not glued back on to the body. It is also missing a portion of the ventral margin of the anterior epiphysis and vertebral body. The neural spine and transverse processes are anteroposteriorally broad. The left prezygapophysis has a generally bulbuous appearance and lacks well defined articular surfaces. The left transverse process is somewhat spongy but is convex instead of concave. This may represent the ultimate or penultimate thoracic vertebra. The tip of the transvers process is not as large as that of vertebra B. A small piece (10 cm long, 3–4 cm wide) of the epiphysis of the next most posterior vertebra is attached to the posterior surface of the caudal end of the vertebra.

**Figure 3:**
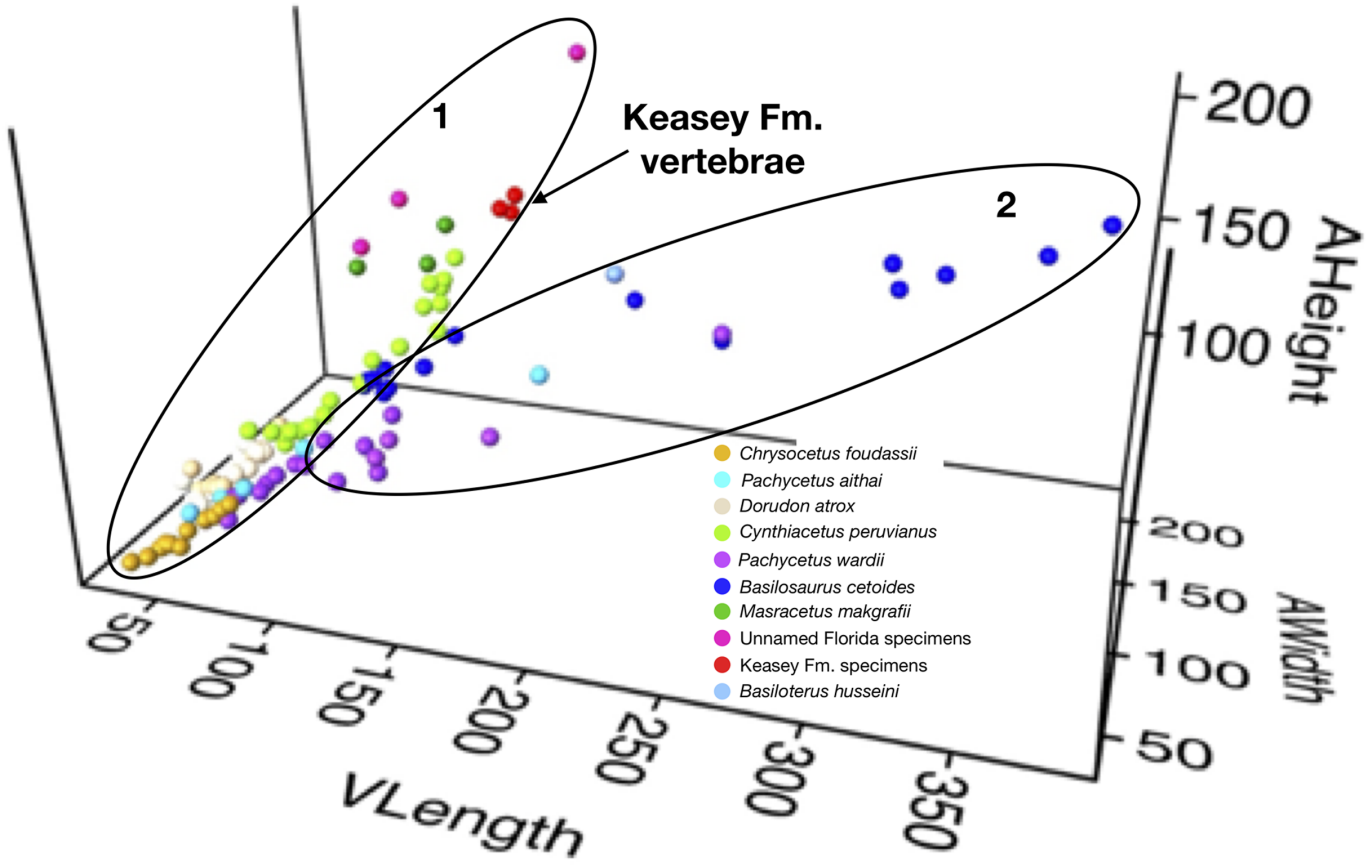
Basilosaurid vertebrae plot. It shows a plot of the dimensions of various basilosaurid thoracic vertebrae. This three-dimensional plot of ventral length, anterior height, and anterior width of the vertebral bodies separates them out based on both size and shape. Thoracic vertebrae in sequence from a single individual show that the thoracic vertebrae increase in all dimensions from about T5 to the ultimate thoracic vertebra in all taxa where such sequences are known. In addition, the different taxa separate into three clusters based on size and shape. Most of the taxa in this figure (*Ancalecetus*, *Chrysocetus*, *Cynthiacetus*, *Dorudon*, *Stomerius*, and *Zygorhiza*) have proportional vertebral centra where length is similar to width and/or height of the vertebral body. These are grouped into Cluster 1. Cluster 2 includes taxa of various sizes that have vertebral bodies much longer than they are wide or high. This cluster includes: *Basilosaurus*, *Basiloterus*, and *Pachycetus*.

**Additional large equidimensional basilosaurid vertebrae**: Several additional specimens of basilosaurid vertebrae are known that fall outside the expected range of variation for currently named genera and species. All of those noted here are larger than comparable vertebrae in *Cynthiacetus peruvianus* (Martínez-Cáceres, Lambert & Muizon, 2017). Like *Cynthiacetus* and *Masracetus* (Gingerich, 2007), they all lack elongation of the vertebral bodies seen in the trunk vertebrae of *Basilosaurus* and *Basiloterus* (Gingerich et al., 1997, Kellogg, 1936) and to some extent *Pachycetus* (Gol’din & Zvonok, 2013, Uhen, 1999, 2001; Gingerich & Zouhri, 2015; van Vliet et al., 2020). See Fig. 3 for a comparison of the sizes and shapes of the trunk vertebrae of these and other basilosaurid taxa. Table 2 includes other named basilosaurid genera along with morphological, temporal, and geographic information for each genus.

**Table 2:** Basilosaurid genera.

Genus	Size	Length	Processes	FAD	LAD	Region
*Ancalecetus*	small	short	wide	Pri	Pri	Tethys
*Basilosaurus*	large	long	wide	Bar	Pri	Tethys/WNA
*Basiloterus*	large	longish	wide	Bar	Bar	Tethys
*Chrysocetus*	small	short	wide	Bar	Pri	WNA/W. Af.
*Cynthiacetus*	large	short	wide	Pri	Pri	WNA/ESP
*Dorudon*	medium	short	wide	Pri	Pri	Tethys/WNA
*Masracetus*	large	short	wide	Pri	Pri	Tethys
*Ocucajea*	small	?	?	Bar	Bar	ESP
*Pachycetus*	large	longish	v. wide	Bar	Pri	Tethys/WNA
*Saghacetus*	small	short	wide	Pri	Pri	Tethys
*Stromerius*	small	short	wide	Pri	Pri	Tethys
Sulaimanitherium*	large	long	wide	Bar	Bar	Tethys
*Supayacetus*	small	?	?	Bar	Bar	ESP
*Zygorhiza*	medium	short	wide	Pri	Pri	WNA

**Notes:**

* *Sulaimanitherium dhanotri* (Malkani et al., 2013) is here designated a *nomen dubium*. See the Results: Taxonomic Note section for details.

Currently known genera of basilosaurid archaeocetes with morphological parameters, temporal range, and geographic region(s) they are known from. Bar, Bartonian; Pri, Priabonian; WNA, Western North Atlantic; W. Af., West Africa; ESP, Eastern South Pacific; Tethys, Tethys Sea.

USNM 776: One lumbar vertebra. Identified as *Pontogeneus brachyspondylus* by Kellogg (1936) and as *Cynthiacetus maxwelli* by Uhen (2005). Locality data associated with the specimen indicate that it was collected from a “marl bank, Patuxent River, Maryland” by T. J. Stone and dated March 8, 1882. Kellogg (1936) seemed skeptical of this and stated that it was from the upper Jackson Formation, precise locality unknown. No late Eocene outcrops are known in the bed of the Patuxent River in Maryland, so the origin of this specimen remains a mystery. It is best identified as Basilosauridae indet.

USNM 510830: One lumbar vertebra. This specimen was discovered in the collection and thus has no associated collecting information. It is best identified as Basilosauridae indet.

FGS V7235: Three thoracic, two lumbar vertebrae, and four undetermined vertebrae with four partial ribs. Collected from the Crystal River Formation, Priabonian, Lafayette County, Florida. Uhen (2005) identified this specimen as *Cynthiacetus maxwelli*, but it is probably too large to be included in that species and is best identified as Basilosauridae indet.

FGS V3888: One lumbar vertebra, missing both anterior and posterior epiphyses. Collected from the Florida Lime Company Pit #2, two miles south of Ocala, Marion County, Florida. This exposure represents the type locality of the Priabonian aged Ocala Limestone (Cooke, 1915).

**Differential identification:** The age (Priabonian), environment of deposition (deep marine), and overall construction of the vertebrae (mammalian) indicate that the vertebrae came from a cetacean. During the Priabonian, with the exception of a single protocetid from the earliest Priabonian of Egypt (Gingerich, Antar & Zalmout, 2019), the only known cetaceans are either members of the Family Basilosauridae; stem Neoceti (e.g., Kekenodontidae) currently understood to be represented by a ghost lineage in the Priabonian (Hernández-Cisneros & Tsai, 2016; Uhen, 2018); and stem Mysticeti (Muizon et al., 2019; Fordyce & Marx, 2018). No Odontoceti are currently known from the Priabonian, and those from the earliest Oligocene are small. Thus, they will be excluded from the following comparison. Also, given that no stem Neoceti (as currently understood) are known from the Priabonian, there are no specimens available for comparison, and they too will be excluded from consideration. This leaves only basilosaurid archaeocetes and stem Mysticeti as potential taxonomic identities for the vertebrae.

NWMNH 2151 cannot be identified as any of the currently named basilosaurids based on its size and the shape of the vertebral centra. It is larger than *Chrysocetus*, *Dorudon*, *Ocucajea*, *Saghacetus*, *Stromerius*, *Supayacetus*, and *Zygorhiza*. The vertebral centra lack the elongation seen in *Basilosaurus*, *Basiloterus*, and *Pachycetus*. The centra are relatively longer than those of *Masracetus*, and it is slightly smaller as well. Finally, the centra of NWMNH 2151 are similarly proportioned to those of *Cynthiacetus* but are notably larger in all dimensions. See Fig. 3 for a comparison of the sizes and shapes of basilosaurid trunk vertebral centra.

## Results

**Basilosaurid morphological affinities**: The size of the vertebrae does not exclusively determine whether the vertebrae belong to a basilosaurid or a stem mysticete. While most of the earliest mysticetes such as *Coronodon* (Geisler et al., 2017) and *Mystacodon* (Muizon et al., 2019) are too small to have had thoracic vertebrae the size of those of NWMNH 2151, *Llanocetus denticrenatus* (Mitchell, 1989) is quite large, and may have had thoracic vertebrae similar in size to those of NWMNH 2151 (Fordyce & Marx, 2018). That said, *Llanocetus* is the only early mysticete known that could possibly be the size of NWMNH 2151, and none are known from the entire Northern Hemisphere. By contrast, the morphological details of the shapes of the vertebrae indicate basilosaurid affinities. First, the texture of the bone surface displays several tiny vascular foramina. This feature can be seen in other large basilosaurids such as *Pachycetus* (Gol’din & Zvonok, 2013, Uhen, 1999; van Vliet et al., 2020) and some specimens of *Cynthiacetus* and *Basilosaurus*. Second, the neural arches, neural spines, and transverse processes of the vertebrae are anteroposteriorly broad. Neural arches, neural spines, and transverse processes of Mysticeti tend to be more gracile. Finally, the transverse processes of the vertebrae angle distinctly ventrally. All crown Neoceti have transverse processes that project from the bodies of their respective vertebrae at almost 90°. However, both stem Odontoceti (Boessenecker, Ahmed & Geisler, 2017) and stem Mysticeti (Boessenecker & Fordyce, 2015) share this feature with basilosaurids. These features together indicate that these three thoracic vertebrae belong to a basilosaurid archaeocete.

Figure 3 shows a plot of the dimensions of various basilosaurid thoracic vertebrae. This three-dimensional plot of ventral length, anterior height, and anterior width of the vertebral bodies separates them out based on both size and shape. Thoracic vertebrae in sequence from a single individual show that the thoracic vertebrae increase in all dimensions from about T5 to the ultimate thoracic vertebra in all taxa where such sequences are known. In addition, the different taxa separate into three clusters based on size and shape. Most of the taxa in Fig. 3 (*Ancalecetus*, *Chrysocetus*, *Cynthiacetus*, *Dorudon*, *Stomerius*, and *Zygorhiza*) have proportional vertebral centra where length is similar to width and/or height of the vertebral body. These are grouped into Cluster A. Cluster B includes taxa of various sizes that have vertebral bodies much longer than they are wide or high. This cluster includes: *Basilosaurus*, *Basiloterus*, and *Pachycetus*.

NWMNH 2151 plots with Cluster A, but it is larger than the largest named taxa in this group, *Cynthiacetus peruvianus* and *Masracetus markgrafi*. Interestingly, a single vertebra from Florida, FGS V-3888 plots in the same cluster along the same trajectory but is significantly larger still. While this is a lumbar vertebra and not a thoracic vertebra like the others, basilosaurids tend to have posterior thoracic vertebrae that are similar in size to lumbar vertebrae. Both the Keasey specimen and this Florida specimen suggest there are large basilosaurid taxa with proportional vertebrae that remain undescribed due to a small number of specimens and lack of associated cranial material.

**Taxonomic Note:** The holotype specimen of *Sulaimanitherium dhanotri* (Malkani et al., 2013) is a series of vertebrae of a single individual of a basilosaurid with elongate trunk vertebral bodies. Unfortunately, the diagnosis of this genus and species does not differentiate this taxon from other basilosaurids with elongate vertebrae. The original description does not include measurements of the vertebrae to help identify it as or distinguish it from similar forms. In addition, it is not clear that the holotype is properly reposited in a museum. For these reasons, both the genus *Sulaimanitherium* and species *Sulaimanitherium dhanotri* are considered *nomina dubia*, and the holotype specimens MSID-1 to MSID-100 are here identified as Basilosauridae indet.

## Discussion

The only other occurrences of cetaceans from the Pacific Ocean that have been suggested to be from the Eocene are as follows. Kellogg (1936, pp. 258–260) listed a single lumbar vertebra (Canadian Geological Survey 8748; now Canadian Museum of Nature FV 8748) discovered near Escalante Point, Vancouver Island, British Columbia (PBDB collection 55764). At that time, Kellogg noted that Ralph B. Stewart concluded that the beds from which the vertebrae was derived was probably Late Eocene or Early Oligocene and likely to be similar in age to the “Lincoln horizon of western Washington”, which is now known as the Lincoln Creek Formation. Jeletzky (1954) identified the rocks at Escalante Point as part of his “Division A” suite from the Hesquiat-Nootka area. These rocks were later identified as belonging to the Escalante Formation (Bancroft, 1937; Cameron, 1980). The Escalante Formation has consistently been thought to be of Refugian age, equivalent to the “Lincoln” aged beds of Washington (Jeletzky, 1954, 1973, 1975). Only recently have opinions shifted to place the Escalante formation in the Eocene (Bartonian–Priabonian) based on microfossil evidence (Cameron, 1980; Nesbitt, 2018). The morphology of this vertebra as depicted by Kellogg (1936, p. 259, Fig. 85) is consistent with it being a basilosaurid archaeocete, and the reassignment of the rocks from Oligocene to Eocene also supports this assignment. Measurements of this specimen are listed in Table 1 and show that it is part of the equidimensional group of vertebrae.

Another cetacean specimen has been noted in the literature from the Middle Fork of the Satsop River, Mason County, Washington. This specimen, UWBM 87312 (informally known as the “Satsop Whale”) was collected from the Lincoln Creek Formation (Kiel, 2008), which in this area has been suggested to be Priabonian (Late Eocene) or Rupelian (Early Oligocene) in age. This specimen has not been described formally, but it appears to be an edentulous mysticete (Kiel, 2008). More stratigraphic work will be needed to confirm its age, and more preparation and study will be needed to confirm its taxonomic identity.

This new discovery of basilosaurid archaeocete vertebrae from the Priabonian (late Eocene) middle Keasey Formation clearly indicates that basilosaurids occurred in the North Pacific Ocean during this time, like they did in almost all other ocean basins. These vertebrae do not match the size and morphology of any currently named basilosaurid, but they are most similar overall to *Cynthiacetus*, which is currently known from the Gulf Coast of North America and the west coast of Peru during the Priabonian (late Eocene). In addition, several other vertebrae or groups of vertebrae from the Gulf Coast have been identified as basilosaurids, but they too do not belong to any currently named species. This indicates that the diversity of Priabonian basilosaurids was considerably higher than previously known, even in areas where they are much more common like the Gulf Coast. The paucity of basilosaurid specimens along the Pacific Coast of North America in rocks of ages where they are well known in other parts of the world might reflect environmental control. For example, since Eocene cetaceans elsewhere in the world are more often found in shallow water pelagic environments, suggesting a preference for shallow water. Thus, the bathyal environmental setting may have been father offshore than they normally preferred.

It could also be suggested that dilution of fossil remains via rapid and massive accumulations of sediments may be an argument that cetaceans certainly occur in the North Pacific, and that such remains are so dispersed they simply have not been found. Similarly, inland (non-coastal) exposures of formations such as the Keasey in the Pacific Northwest are heavily vegetated, limiting exposures. Nevertheless, there are sufficient exposures in, quarries, roadcuts, and along streams, that vertebrate material would be present if it occurred in any quantity.

We may argue that marginal continental sedimentation around the entire Pacific Basin would be characterized by formations overall of similar thicknesses to those in the NE Pacific- and that sediment dilution, if a problem—should be a characteristic of the entire Pacific rim. The Keasey is about 700 m thick. One would expect that exceptionally rapid sediment accumulation would be one in which primary sediment structures would predominate. Instead, throughout the middle member of the Keasey, the lithologies are thoroughly bioturbated, suggesting modest depositional rates. The formation was deposited over a time interval of about 3 million years, also consistent with such modest depositional rates. We suggest that the marked paucity of Eocene vertebrates from the North Pacific, rather than being an artifact from lack of exposure or sediment dilution, mostly likely does reflect an actual paucity of such mammals in the North Pacific.

## Conclusions

Three posterior thoracic vertebrae found in the Priabonian Middle Member of the Keasey Formation near Vernonia, Oregon are here identified as the first definitive basilosaurid archaeocete specimen from the North Pacific Ocean basin. These vertebrae demonstrate that while rare, basilosaurids were indeed present in the North Pacific, and suggest that perhaps their rarity is due to the scarcity of shallower water continental shelf deposits in this region rather than their true absence from this region. The paucity of late Eocene basilosaurids in the North Pacific might have resulted in part from the comparatively cool water temperatures which they may not have favored.

## Supplemental Information

10.7717/peerj.9809/supp-1 Supplemental Information 1 Keasey basilosaurid additional measurements and references.

## Abbreviations

GSC: Geological Survey of Canada, Ottawa, Canada
MSID: M. Shahid Ishaq Dhanotr
NWMNH: Northwest Museum of Natural History, Portland, OR
PBDB: Paleobiology Database
UF: Florida Museum of Natural History, University of Florida, Gainesville, FL
USNM: United States National Museum, Washington, DC
UWBM: University of Washington, Burke Museum, Seattle, Washington

## Additional Information and Declarations

### Field Study Permissions

The following information was supplied relating to field study approvals (i.e., approving body and any reference numbers): The late Ralph Keasey, former land manager for the Keasey Family Corporation, gave permission in the late 1980s for us to conduct our field work and obtain the whale specimen.

### Data Availability

The following information was supplied regarding data availability: The specimen described in this manuscript is NWMNH 2151, three sequential posterior thoracic vertebrae. The NWMNH, Northwest Museum of Natural History is in Portland, OR. The complete measurements of the vertebrae and supplementary bibliographic information on basilosaurid archaeocetes from the Paleobiology Database are available in the Supplemental File.
