# Supplementary material for: A basilosaurid archaeocete (Cetacea, Pelagiceti) from the Late Eocene of Oregon, USA
Source: PeerJ. 2020 Oct 2;8:e9809. doi: 10.7717/peerj.9809 (PMC7534682; doi:10.7717/peerj.9809)
Supplement: Supplemental Information 1 [file peerj-08-9809-s001.docx]

Oregon basilosaurid Supplementary Information

**Measurements**

All measurements are in millimeters.

| **Genus** | **Species** | **Museum** | **No.** | **Vert** | **AW** | **AH** | **VL** | **DL** | **PW** | **PH** |
| --- | --- | --- | --- | --- | --- | --- | --- | --- | --- | --- |
| *Cynthiacetus* | *maxwelli* | USNM | 776 | L | 211 | 179 | 216 | 214 | ~216 | ~186 |
|  |  | USNM | 510830 | L | ~180 | ~151 | 130+ | 138+ | 177 | ~157 |
|  |  | FGS | V3888 | L | ~218 | 201 | 154 | - | - | - |
| *Cynthiacetus* | *maxwelli* | FGS | V7235 | TB | 173 | ~144 | 89.2 | 97 | 170 | 140.6 |
|  |  | Oregon |  | TA | 176 | 142 | 139 | 138 | 169 | 146 |
|  |  | Oregon |  | TB | 171 | 145 | 136 | 144 | 181 | 150 |
|  |  | Oregon |  | TC | 178 | ~149 | ~140 | 148 | 185 | 156 |
|  |  |  |  |  |  |  |  |  |  |  |
|  |  |  |  |  |  |  |  |  |  |  |
|  |  |  |  |  |  |  |  |  |  |  |
|  |  |  |  |  |  |  |  |  |  |  |

(Abel, 1906; Adnet et al., 2010; Andrews, 1904, 1906; Anonymous, 1952; Applegate, 1969; Beadnell, 1905; Benton et al., 2005; Blanckenhorn, 1903; Bogachev, 1959; Brandt, 1873a, b; Breard and Stringer, 1995; Buckley, 1846; Buono et al., 2016; Cooke, 1915; Cozzuol, 1988; Dart, 1923; Dockery and Johnston, 1986; Dockery et al., 2003; Dubrovo and Kapelist, 1973; El-kheir et al., 2013; Élouard, 1966; Emmer and Dunn, 1996; Fedorowski, 1912; Field et al., 2011; Fordyce and Hiller, 2014; Fordyce and Roberts, 2009; Fostowicz-Frelik, 2003; Geisler et al., 2005; Gibbes, 1845; Gingerich, 1992, 2007, 2010; Gingerich and Uhen, 1996; Goedert, 1988; Gol'din et al., 2012; Harlan, 1834; Hilgard, 1860; Holman, 2001; Insacco, 2014; Kellogg, 1936; Koch, 1899; Lancaster, 1982, 1986; Leidy, 1852; Lucas, 1900; Martínez-Cáceres and de Muizon, 2011; McPherson and Manning, 1986; Moore and Brown, 1969; Morgan, 1978; Palmer, 1939; Pilleri, 1991; Reel, 1972; Reichenbach, 1847; Sanders and Katuna, 2000; Schouten, 2011; Sellards, 1916; Smith et al., 2013; Stromer, 1902, 1903; Sullivan, 1948; Uhen, 2004, 2005, 2013; Uhen and Gingerich, 2001; Underwood et al., 2011; van Vliet and el Khair, 2010; van Vliet et al., 2019; Voss et al., 2019; Wallace, 1963; Weems and Brown, 2017; Westgate, 2008; Zalmout et al., 2012; Zalmout et al., 2011; Zalmout and Gingerich, 2012; Zalmout et al., 2000; Zouhri et al., 2019a; Zouhri et al., 2014; Zouhri et al., 2019b)

**Figure 3 data**

Measurements are in millimeters.

| **Specimen/ Vertebra** | **Ventral Length** | **Anterior Width** | **Anterior Height** | **Taxon** |
| --- | --- | --- | --- | --- |
| UWBM 82916 T10 | 65.5 | 95.4 | 85.8 | *Sitsquayk cornishorum* |
| UWBM 82916 T11 | 78.7 | 96.4 | 82.1 | *Sitsquayk cornishorum* |
| UWBM 82916 T12 | 77.5 | 92.3 | 83.3 | *Sitsquayk cornishorum* |
| UWBM 82916 T13 | 83.8 | 107.1 | 84.57 | *Sitsquayk cornishorum* |
| UWBM 82916 T14 | 95.5 | 103.4 | 92.48 | *Sitsquayk cornishorum* |
| NWMNH 2151 A | 139 | 176 | 142 | *Basilosauridae indet.* |
| NWMNH 2151 B | 136 | 171 | 145 | *Basilosauridae indet.* |
| NWMNH 2151 C | 140 | 178 | 149 | *Basilosauridae indet.* |
| NSFM 4470 A | 119 | 80 | 65 | *? Eocetus* |
| USNM 310633 TA | 59.5 | 66.1 | 47 | *Basilotritus wardii* |
| USNM 449548 TB | 89.2 | 76.6 | 65 | *Basilotritus wardii* |
| USNM 310633 TC | 102 | 78 | 59.1 | *Basilotritus wardii* |
| GSP-UM 3193 | 300 | 168 | 141 | *Basilosaurus drazindai* |
| GSP-UM 3190 | 195 | 144 | 133 | *Basiloterus hussaini* |
| Bouj 27 | 240 | 140 | 115 | *Eocetus schweinfurthi* |
| FSAC Bouj-6 T1 | 50 | 54 | 49 | *Platyosphys aithai* |
| FSAC Bouj-6 T2 | 57 | 66 | 51 | *Platyosphys aithai* |
| FSAC Bouj-6 T3 | 67 | 67 | 56 | *Platyosphys aithai* |
| FSAC Bouj-6 T4 | 84 | 86 | 67 | *Platyosphys aithai* |
| FSAC Bouj-6 T12 | 175 | 111 | 101 | *Platyosphys aithai* |
| FSAC Bouj-2 T2 | 34 | 35 | 35 | *Chrysocetus fouadassii* |
| FSAC Bouj-2 T4 | 39 | 39 | 36 | *Chrysocetus fouadassii* |
| FSAC Bouj-2 T5 | 40 | 46 | 36 | *Chrysocetus fouadassii* |
| FSAC Bouj-2 T6 | 42 | 47 | 38 | *Chrysocetus fouadassii* |
| FSAC Bouj-2 T7 | 44 | 47 | 38 | *Chrysocetus fouadassii* |
| FSAC Bouj-2 T8 | 48 | 48 | 37 | *Chrysocetus fouadassii* |
| FSAC Bouj-2 T9 | 50 | 51 | 43 | *Chrysocetus fouadassii* |
| FSAC Bouj-2 T10 | 55 | 56 | 46 | *Chrysocetus fouadassii* |
| FSAC Bouj-2 T11 | 58 | 58 | 47 | *Chrysocetus fouadassii* |
| FSAC Bouj-2 T13 | 61 | 59 | 50 | *Chrysocetus fouadassii* |
| FSAC Bouj-2 T14 | 65 | 59 | 53 | *Chrysocetus fouadassii* |
| NCSM 11284 T1 | 51 | 62.3 | 41.5 | *Basilotritus wardii* |
| NCSM 11284 T2 | 63.3 | 59.4 | 45.2 | *Basilotritus wardii* |
| NCSM 11284 T3 | 63.7 | 67.1 | 52 | *Basilotritus wardii* |
| NCSM 11284 T4 | 60.3 | 64.2 | 48 | *Basilotritus wardii* |
| NCSM 11284 T5 | 58.4 | 68 | 50.5 | *Basilotritus wardii* |
| NCSM 11284 T6 | 74.3 | 66.8 | 56 | *Basilotritus wardii* |
| NCSM 11284 T7 | 74 | 72.2 | 56.7 | *Basilotritus wardii* |
| NCSM 11284 T8 | 78.2 | 73 | 60.6 | *Basilotritus wardii* |
| NCSM 11284 T9 | 80.5 | 84.2 | 60.2 | *Basilotritus wardii* |
| NCSM 11284 T10 | 89.5 | 93 | 67.9 | *Basilotritus wardii* |
| NCSM 11284 T11 | 111 | 85.9 | 72.5 | *Basilotritus wardii* |
| NCSM 11284 T12 | 118.1 | 97 | 81.8 | *Basilotritus wardii* |
| UM 101216 T1 | 38.6 | 75.2 | 55.9 | *Dorudon atrox* |
| UM 101216 T3 | 46.8 | 66.9 | 52.2 | *Dorudon atrox* |
| UM 101216 T4 | 49.8 | 72.3 | 52.7 | *Dorudon atrox* |
| UM 101216 T5 | 49.5 | 69.7 | 52.5 | *Dorudon atrox* |
| UM 101216 T6 | 49 | 68.4 | 52.3 | *Dorudon atrox* |
| UM 101216 T8 | 53.6 | 68.3 | 53.4 | *Dorudon atrox* |
| UM 101216 T9 | 56.3 | 69.2 | 54.3 | *Dorudon atrox* |
| UM 101216 T10 | 58.7 | 77.2 | 56.7 | *Dorudon atrox* |
| UM 101216 T11 | 62.4 | 82.1 | 63.4 | *Dorudon atrox* |
| UM 101216 T12 | 64 | 85.2 | 63.5 | *Dorudon atrox* |
| UM 101216 T13 | 65.8 | 85 | 63.8 | *Dorudon atrox* |
| UM 101216 T14 | 68.3 | 93 | 68.6 | *Dorudon atrox* |
| UM 101216 T15 | 68.9 | 93.7 | 68.1 | *Dorudon atrox* |
| UM 101216 T16 | 71.3 | 90.1 | 73.2 | *Dorudon atrox* |
| MNHN.F.PRU10 T1 | 62.7 | 81.2 | 73.9 | *Cynthiacetus peruvianus* |
| MNHN.F.PRU10 T2 | 75.3 | 86.3 | 71.8 | *Cynthiacetus peruvianus* |
| MNHN.F.PRU10 T3 | 81.7 | 87.1 | 73.7 | *Cynthiacetus peruvianus* |
| MNHN.F.PRU10 T4 | 75.7 | 86 | 67.8 | *Cynthiacetus peruvianus* |
| MNHN.F.PRU10 T5 | 79.8 | 84.3 | 74.8 | *Cynthiacetus peruvianus* |
| MNHN.F.PRU10 T6 | 72.3 | 82.3 | 74.2 | *Cynthiacetus peruvianus* |
| MNHN.F.PRU10 T7 | 87.3 | 86.4 | 77.1 | *Cynthiacetus peruvianus* |
| MNHN.F.PRU10 T8 | 91 | 91.2 | 80.5 | *Cynthiacetus peruvianus* |
| MNHN.F.PRU10 T9 | 91.3 | 97 | 84 | *Cynthiacetus peruvianus* |
| MNHN.F.PRU10 T10 | 99.9 | 107.4 | 88.4 | *Cynthiacetus peruvianus* |
| MNHN.F.PRU10 T11 | 102 | 114.3 | 95.7 | *Cynthiacetus peruvianus* |
| MNHN.F.PRU10 T12 | 111 | 121.4 | 99.8 | *Cynthiacetus peruvianus* |
| MNHN.F.PRU10 T13 |  | 131 | 106.4 | *Cynthiacetus peruvianus* |
| MNHN.F.PRU10 T14 | 123.3 | 132 | 104 | *Cynthiacetus peruvianus* |
| MNHN.F.PRU10 T15 | 116.1 | 135 | 112.7 | *Cynthiacetus peruvianus* |
| MNHN.F.PRU10 T16 | 124.4 | 133.2 | 115.8 | *Cynthiacetus peruvianus* |
| MNHN.F.PRU10 T17 | 119.5 | 135.1 | 123.2 | *Cynthiacetus peruvianus* |
| MNHN.F.PRU10 T18 | 122.6 | 138.4 | 120.7 | *Cynthiacetus peruvianus* |
| MNHN.F.PRU10 T19 | 123.8 | 138.7 | 124 | *Cynthiacetus peruvianus* |
| MNHN.F.PRU10 T20 | 126.2 | 145.2 | 131.5 | *Cynthiacetus peruvianus* |
| USNM 4675 T1 | 92 | 125 | 78 | *Basilosaurus cetoides* |
| USNM 4675 T2 | 102 | 125 | 87 | *Basilosaurus cetoides* |
| USNM 4675 T3 | 112 | 107 | 88 | *Basilosaurus cetoides* |
| USNM 4675 T4 | 104.5 | 109 | 90 | *Basilosaurus cetoides* |
| USNM 4675 T5 | 102 | 109 | 89 | *Basilosaurus cetoides* |
| USNM 4675 T6 | 108.5 | 110 | 84 | *Basilosaurus cetoides* |
| USNM 4675 T7 | 122 | 120 | 93 | *Basilosaurus cetoides* |
| USNM 4675 T8 | 128 | 138 | 100 | *Basilosaurus cetoides* |
| USNM 4675 T9 | 196 | 167 | 113 | *Basilosaurus cetoides* |
| USNM 4675 T10 | 236 | 156 | 105 | *Basilosaurus cetoides* |
| USNM 4675 T11 | 302 | 177 | 127 | *Basilosaurus cetoides* |
| USNM 4675 T12 | 319 | 183 | 133 | *Basilosaurus cetoides* |
| USNM 4675 T13 | 357 | 184 | 145 | *Basilosaurus cetoides* |
| USNM 4675 T14 | 378 |  |  | *Basilosaurus cetoides* |
| USNM 4675 T15 | 379 | 186.5 | 159 | *Basilosaurus cetoides* |
| USNM 4679 T2 | 43 |  |  | *Zygorhiza kochii* |
| USNM 4679 T3 | 51.5 |  | 50.8 | *Zygorhiza kochii* |
| USNM 4679 T4 | 52 |  |  | *Zygorhiza kochii* |
| USNM 4679 T5 | 55.8 | 68.5 | 50.3 | *Zygorhiza kochii* |
| USNM 4679 T6 | 56.5 | 81 | 51 | *Zygorhiza kochii* |
| USNM 4679 T7 | 58 | 78 | 52.5 | *Zygorhiza kochii* |
| USNM 4679 T8 | 59.5 | 73.5 | 57.8 | *Zygorhiza kochii* |
| USNM 4679 T9 | 63 | 78 | 58.5 | *Zygorhiza kochii* |
| USNM 4679 T10 |  | 81.2 | 59.6 | *Zygorhiza kochii* |
| USNM 4679 T11 | 68.6 | 78.5 | 66 | *Zygorhiza kochii* |
| USNM 4679 T12 | 66.5 | 78.5 | 66.8 | *Zygorhiza kochii* |
| USNM 4679 T13 | 68.5 | 77.7 | 66.8 | *Zygorhiza kochii* |
| USNM 4679 T14 | 68.3 | 79 | 65 | *Zygorhiza kochii* |
| USNM 4679 T15 | 69.8 | 79.8 | 66.2 | *Zygorhiza kochii* |
| CGM 42290 T1 | 35.7 | 72.6 | 51.2 | *Ancalecetus simonsi* |
| CGM 42290 T2 | 42.8 | 67.1 | 46.8 | *Ancalecetus simonsi* |
| CGM 42290 T3 | 46.4 | 64.5 |  | *Ancalecetus simonsi* |
| CGM 42290 T4 | 46.5 | 65.7 | 50.3 | *Ancalecetus simonsi* |
| CGM 42290 T5 | 48.2 | 67.9 | 49.5 | *Ancalecetus simonsi* |
| CGM 42290 T6 | 51.2 | 68.5 | 50.3 | *Ancalecetus simonsi* |
| CGM 42290 T7 | 54.5 |  | 51.9 | *Ancalecetus simonsi* |
| CGM 42290 T11 | 55.2 | 84.7 | 60.4 | *Ancalecetus simonsi* |
| CGM 42290 T12 | 58.9 | 79.9 | 61.2 | *Ancalecetus simonsi* |
| CGM 42290 T14 | 59.5 | 84 | 65 | *Ancalecetus simonsi* |
| CGM 42290 T15 | 60.9 | 81.7 | 67.6 | *Ancalecetus simonsi* |
| CGM 42290 T16 | 60 | 78.9 |  | *Ancalecetus simonsi* |
| UM 100140 T3 | 35 |  | 35 | *Stomerius nidensis* |
| UM 100140 T4 | 33 |  | 35 | *Stomerius nidensis* |
| UM 100140 T5 | 36.3 |  | 35 | *Stomerius nidensis* |
| UM 100140 Tn | 51.3 | 59.3 | 50 | *Stomerius nidensis* |
| NMNH-P OF-2096 TA | 119 | 79 | 72 | *Basilotritus uheni* |
| NMNH-P OF-2096 TA | 120 | 89 | 75 | *Basilotritus uheni* |
| NMNH-P OF-2096 TA | 159 | 98 | 78 | *Basilotritus uheni* |
| FGS V-4894 Lx | 82.8 | 148 | 130 | *Basilosauridae indet.* |
| FGS V-3888 Lx | 154 | 218 | 201 | *Basilosauridae indet.* |
| FGS V-7235 Tb | 89.2 | 173 | 143.6 | *Basilosauridae indet.* |
| SMNS 11414 Tx1 | 107 | 160 | 121 | *Masracetus markgrafi* |
| SMNS 11414 Tx2 | 111 | 171 | 135 | *Masracetus markgrafi* |
| SMNS 11414 Tx3 | 130 |  | 135 | *Masracetus markgrafi* |
| BSPM 1904.XII.135 T12 | 78.9 | 150.9 | 119.6 | *Masracetus markgrafi* |

References Cited

Abel, O., 1906, Über den als Beckengürtel von *Zeuglodon* beschriebenen Schultergürtel eines Vogels aus dem Eocän von Alabama: Centralblatt für Mineralogie, Geologie und Paläontologie, v. 15, p. 450–458.

Adnet, S., Cappetta, H., and Tabuces, R., 2010, A Middle–Late Eocene vertebrate fauna (marine fish and mammals) from southwestern Morocco; preliminary report: age and palaeobiogeographical implications: Geological Magazine, v. 147, no. 6, p. 860–870.

Andrews, C. W., 1904, Further notes on the mammals of the Eocene of Egypt: Geological Magazine, v. 1, p. 211–215.

-, 1906, A Descriptive Catalogue of the Tertiary Vertebrata of Fayum, Egypt, London, British Museum of Natural History, 324 p.:

Anonymous, I. M., 1952, Basilosaurus cetoides from Crawford County, Georgia: Georgia Mineral Society Newsletter, v. 5, no. 2, p. 54.

Applegate, S. P., 1969, Digging fossil whales in Mississippi with southern hospitality: Los Angeles County Museum of Natural History Quarterly, v. 8, no. 3, p. 26–31.

Beadnell, H. J. L., 1905, The Topography and Geology of the Fayum Province of Egypt, Cairo, Survey Department Egypt, 101 p.:

Benton, M. J., Cook, E., and Hooker, J. J., 2005, Mesozoic and Tertiary Fossil Mammals and Birds of Great Britain, London, Joint Nature Conservation Committee (Geological Conservation Review), Geological Conservation Review Series, 215 p.:

Blanckenhorn, M., 1903, Neue geologisch-stratigraphische Beobachtungen in Aegypten: Sitzungsberichte der mathematisch-physikalischen Classe der Akademie der Wissenschaften, v. 22, p. 351–433.

Bogachev, V. V., 1959, [Remains of a cetacean from the Oligocene of Tsimlyanskaya Station]: Akademia Nauk USSR, Kiev. Instytut mineral'nykh resursiv, Trudy, v. 1, p. 40–42.

Brandt, J. F., 1873a, Über bisher in Russland gefundene Reste von Zeuglodonten: Melanges biologiques Bulletin de l'Academie imperials des Sciences de St. Petersbourg, v. 9, p. 111–112.

-, 1873b, Untersuchungen über die fossilen und subfossilen cetaceen Europa's: Mémoires de L'Académie Impériale des Sciences de Saint-Petersbourg, Series 7, v. 20, no. 1, p. 1–372.

Breard, S. Q., Jr., and Stringer, G. L., 1995, Paleoenvironment of a diverse marine vertebrate fauna from the Yazoo Clay (Late Eocene) at Copenhagen, Caldwell Parish, Louisiana: Transactions of the Gulf Coast Association of Geological Societies, v. 45, p. 77–85.

Buckley, S. B., 1846, On the Zeuglodon remains of Alabama: The American Journal of Science and Arts, v. 2, p. 125–131.

Buono, M., Fernández, M., Reguero, M., Marenssi, S., Santillana, S., and Mörs, T., 2016, Eocene basilosaurid whales from the La Meseta Formation, Marambio (Seymour) Island, Antarctica: Ameghiniana, v. 53, no. 3, p. 296-315.

Cooke, C. W., 1915, The age of the Ocala limestone: Department of the Interior US Geological Survey, v. 95-I, p. 107–117.

Cozzuol, M. A., 1988, Comentarios sobre los Archaeoceti (Mammalia: Cetacea), de la Isla Vicecomodoro Marambio, Antártida: Jornadas Argentinas de Paleontologia de Vertebrados, v. 5, p. 32.

Dart, R. A., 1923, The brain of the Zeuglodontidae (Cetacea): Proceedings of the Zoological Society of London, v. 42, no. 33, p. 615–654.

Dockery, D. T., III, and Johnston, J. E., 1986, Excavation of an archaeocete whale, *Basilosaurus cetoides* (Owen), from Madison, Mississippi: Mississippi Geology, v. 6, no. 3, p. 1–10.

Dockery, D. T., III, Starnes, J. E., and Peyton, S., 2003, A largely complete *Basilosaurus* find in the Upper Yazoo Clay in Scott County, Mississippi: Journal of the Mississippi Academy of Science, v. 48, no. 1, p. 42–43.

Dubrovo, I. A., and Kapelist, K. V., 1973, Catalog of Localities of Tertiary Vertebrates of the USSR.

El-kheir, G. A. A., El Anbaawy, M. I., Helal, S. A., and Gibbs, S., 2013, Dentary of *Masracetus markgrafi*, archaeocete in the north of Lake Qaroun, Fayoum Egypt: Journal of American Science, v. 9, no. 12, p. 459-469.

Élouard, P., 1966, Découverte d'un archéocète dans les environs de Kaolack: Notes Africaines : Bulletin d'Information et de Correspondance de l'Institut Français d'Afrique Noire, v. 109, p. 8–10.

Emmer, E. W., and Dunn, D. A., 1996, Stable isotopic composition of a late Eocene archaeocete whale, *Basilosaurus cetoides*, from Wayne County, Mississippi: AAPG Bulletin, v. 80, no. 9, p. 1501.

Fedorowski, J. A., 1912, Zeuglodon-Reste aus dem Kreise Zmijew, Gouvernement Charkow: Arbeiten der Naturforscher-Gesellschaft an der Kaiserlichen Universitat Charkow, v. 45, p. 253-287.

Field, D. J., Racicot, R. A., and Uhen, M. D., 2011, A new marine tetrapod assemblage from the Eocene of Western Sahara: Journal of Vertebrate Paleontology, v. Abstract Supplement, p. 108–109.

Fordyce, R. E., and Hiller, N., 2014, An associated skeleton of juvenile Late Eocnee basilosaurid archaeocete (Cetacea: Archaeoceti) from New Zealand: Society of Vertebrate Paleontology Meeting Program and Abstracts, v. 74, p. 131.

Fordyce, R. E., and Roberts, C. D., 2009, Fossil Pinnipedia and Cetacea, *in* Gordon, D. P., ed., New Zealand Inventory of Biodiversity: Canterbury, New Zealand, Canterbury University Press, p. 553.

Fostowicz-Frelik, L., 2003, An enigmatic whale tooth from the Upper Eocene of Seymour Island, Antarctica: Polish Polar Research, v. 24, no. 1, p. 13–28.

Geisler, J. H., Sanders, A. E., and Luo, Z.-X., 2005, A new protocetid whale (Cetacea: Archaeoceti) from the Late Middle Eocene of South Carolina: American Museum Novitates, v. 3480, p. 1–65.

Gibbes, R. W., 1845, Description of the teeth of a new fossil animal found in the Green Sand of South Carolina: Proceedings of the Academy of Natural Sciences of Philadelphia, v. 2, p. 254–256.

Gingerich, P. D., 1992, Marine mammals (Cetacea and Sirenia) from the Eocene of Gebel Mokattam and Fayum, Egypt: stratigraphy, age and paleoenvironments: The University of Michigan Museum of Paleontology Papers on Paleontology, v. 30, p. 1–84.

-, 2007, *Stromerius nidensis*, new archaeocete (Mammalia, Cetacea) from the Upper Eocene Qasr El-Sagha Formation, Fayum, Egypt: Contributions from the Museum of Paleontology, The University of Michigan, v. 31, no. 13, p. 363–378.

-, 2010, Cetacea, *in* Werdelin, L., and Sanders, W. J., eds., Cenozoic Mammals of Africa: Berkeley, University of California Press, p. 873–899.

Gingerich, P. D., and Uhen, M. D., 1996, *Ancalecetus simonsi*, a new dorudontine archaeocete (Mammalia, Cetacea) from the early late Eocene of Wadi Hitan, Egypt: Contributions from the Museum of Paleontology, The University of Michigan, v. 29, no. 13, p. 359–401.

Goedert, J. L., 1988, A new late Eocene species of Plotopteridae (Aves: Pelecaniformes) from Northwestern Oregon: Proceedings of the California Academy of Sciences, v. 45, no. 6, p. 97–102.

Gol'din, P., Zvonok, E., and Krakhmal'naia, T., 2012, On the problematic Eocene cetacean from Nagornoye site (Kirovograd prov., Ukraine) and the significance of archaeocetes for stratigraphic research: Geolog Ukrainy, v. 1-2, p. 87–93.

Harlan, R., 1834, Notice of fossil bones found in the Tertiary formation of the state of Louisiana: Transactions of the American Philosophical Society Philadelphia, v. 4, no. 12, p. 397–403.

Hilgard, E. W., 1860, Report on the Geology and Agriculture of the State of Mississippi, Jackson, Mississippi, E. Barksdale, 388 p.:

Holman, J. A., 2001, First report of an Eocene reptile fauna from Florida, USA: Palaeovertebrata, v. 30, no. 1-2, p. 1–10.

Insacco, G., 2014, I Cetacei fossili del Museo Civico di Storia Naturale di Comiso (Ragusa): Museologia Scientifica Memoire, v. 13, p. 130-134.

Kellogg, R., 1936, A Review of the Archaeoceti: Carnegie Institution of Washington Special Publication, v. 482, p. 1–366.

Koch, A., 1899, Schwanzwirbel-reste eines ausgestorbenen Cetaceen von Kolozsvár: Földtani Közlöny, v. 19, p. 204–209.

Lancaster, W. C., 1982, A Morphological and Paleoecological Analysis of the Archaeoceti of Montgomery Landing, Louisianacopy]: Auburn University.

-, 1986, The taphonomy of an archaeocete skeleton and its associated fauna, *in* Schiebout, J. A., and van den Bold, W., eds., Montgomery Landing Site, Marine Eocene (Jackson) of Central Louisiana, Proceedings of a Symposium, 36th Annual GCAGS: Baton Rouge, Louisiana, Gulf Coast Association of Geological Societies, p. 119–131.

Leidy, J., 1852, [Description of *Pontogeneus priscus*]: Proceedings of the Academy of Natural Sciences of Philadelphia, v. 6, p. 52.

Lucas, F. A., 1900, The pelvic girdle of zeuglodon *Basilosaurus cetoides* (Owen), with notes on other portions of the skeleton: Proceedings of the United States National Museum, v. 23, p. 327–331.

Martínez-Cáceres, M., and de Muizon, C., 2011, A new basilosaurid (Cetacea, Pelagiceti) from the Late Eocene to Early Oligocene Otuma Formation of Peru: Comptes Rendus Palevol, v. 10, p. 517–526.

McPherson, A. B., and Manning, E. M., 1986, New records of Eocene sea snakes (Pterosphenus) from Louisiana, *in* Schiebout, J. A., and van den Bold, W., eds., Montgomery Landing Site, Marine Eocene (Jackson) of Central Louisiana, Volume Symposium Proceedings: Baton Rouge, Gulf Coast Association of Geological Societies, p. 197-207.

Moore, G. K., and Brown, D. L., 1969, Stratigraphy of the Fort Pillow Test Well, Lauderdale County, Tennessee: Tennessee Division of Geology Report of Investigations, v. 26, p. 1.

Morgan, G. S., 1978, The fossil whales of Florida: The Plaster Jacket, v. 29, p. 1–20.

Palmer, K. V. W., 1939, Basilosaurus in Arkansas: Bulletin of the American Association of Petroleum Geologists, v. 23, no. 8, p. 1228–1229.

Pilleri, G., 1991, Betrachtugen über das gehirn der Archeoceti (Mammalia, Cetacea) aus dem Fayum Ägyptens: Investigations on Cetacea, v. 23, p. 193–211.

Reel, T. W., 1972, The Excavation and Preparation of Two Fossilized Whalescopy]: University of Southern Mississippi.

Reichenbach, H. G. L., 1847, Systematisches, *in* Carus, C. G., ed., Resultate geologischer, anatomischer und zoologischer untersuchungen über das unter den Namen *Hydrarchos* von Dr. A. C. Koch zuerst nach Europa gebrachte und in Dresden augestelte grofse fossile Skelett: Dresden, Arnoldische Buchhandlung, p. 13–15.

Sanders, A. E., and Katuna, M. P., 2000, Proposal for revision of the stratigraphic nomenclature of the Santee Limestone (Eocene) of South Carolina, U.S.A.: Geological Society of America, Southeastern Section, v. 32, no. 2, p. 71.

Schouten, S., 2011, De wervels van Basilosauridae: een overzicht van en een vergelijking met raadselachtige vondsten uit de Noordzee: Cranium, v. 28, no. 2, p. 17-25.

Sellards, E. H., 1916, Fossil vertebrates from Florida: A new Miocene fauna; new Pliocene species; the Pleistocene fauna: Florida State Geological Survey, v. 8, p. 79–119.

Smith, K. M., Hastings, A. K., and Bebej, R. M., 2013, Evolution, dispersal, and habitat preference of Basilosaurus (Mammalia: Cetacea) in the southeastern United States: New evidence from the Eocene of southwest Georgia: Geological Society of America Abstracts with Programs, v. 45, no. 26-27.

Stromer, E., 1902, Bericht über eine von den Privatdozenten Dr. Max Blanckenhorn und Dr. Ernst Stromer von Reichen bach ausgeführte Reisse nach Aegypten: Sitzungsberichten der mathematiche-phys. Akademie der Wissenschaften, v. 32, no. 3, p. 341–352.

-, 1903, Zeuglodon-reste aus dem Oberen Mitteleocän des Fajum: Beiträge zur Paläontologie und Geologie Österreich-Ungarns und des Orients, v. 15, p. 65–100.

Sullivan, J. M., 1948, Some new fossils from the Mississippi Eocene: Journal of the Mississippi Academy of Science, v. 3, p. 153–162.

Uhen, M. D., 2004, Form, function, and anatomy of *Dorudon atrox* (Mammalia, Cetacea): An archaeocete from the Middle to Late Eocene of Egypt: The University of Michigan Museum of Paleontology Papers on Paleontology, v. 34, p. 1–222.

-, 2005, A new genus and species of archaeocete whale from Mississippi: Southeastern Geology, v. 43, no. 3, p. 157–172.

-, 2013, A review of North American Basilosauridae: Alabama Museum of Natural History Bulletin, v. 31, no. 2, p. 1-45.

Uhen, M. D., and Gingerich, P. D., 2001, New genus of dorudontine archaeocete (Cetacea) from the middle-to-late Eocene of South Carolina: Marine Mammal Science, v. 17, no. 1, p. 1–34.

Underwood, C. J., Ward, D. J., King, C., Antar, S. M., Zalmout, I. S., and Gingerich, P. D., 2011, Shark and ray faunas in the Middle and Late Eocene of the Fayum Area, Egypt: Proceedings of the Geologists' Association, v. 122, p. 47-66.

van Vliet, H. J., and el Khair, G. A., 2010, A new Eocene marine mammal site in the Qattara depression (Egypt): Cainozoic Research, v. 7, no. 1-2, p. 73–77.

van Vliet, H. J., Lambert, O., Bosselaers, M., Schulp, A. S., and Jagt, J. W. M., 2019, A Palaeogene cetacean from Maastricht, southern Limburg (The Netherlands): Cainozoic Research, v. 19, no. 1, p. 95-111.

Voss, M., Antar, M. S. M., Zalmout, I. S., and Gingerich, P. D., 2019, Stomach contents of the archaeocete Basilosaurus isis: Apex predator in oceans of the late Eocene: PLoS One, v. 14, no. 1, p. e0209021.

Wallace, W. D., 1963, Alabama boneyard sheds new light on whales: Science Digest, v. 53, no. 4, p. 77–80.

Weems, R. E., and Brown, K. M., 2017, More-complete remains of Procolpochelys charlestonensis (Oligocene, South Carolina), an occurrence of Euclastes (upper Eocene, South Carolina), and their bearing on Cenozoic pancheloniid sea turtle distribution and phylogeny: Journal of Paleontology, v. 91, no. 6, p. 1228-1243.

Westgate, J. W., 2008, Eocene (Jacksonian) estuarine vertebrate faunas from Crowley's Ridge, Arkansas: Geological Society of America. Programs with Abstracts, v. 40, no. 3, p. 3.

Zalmout, I., Antar, M. S. M., Shafy, E. A.-E., Metwally, M. H., Hatab, E.-B. E., and Gingerich, P. D., 2012, Priabonian sharks and rays (Late Eocene: Neoselachii) from Minqar Tabaghbagh in the Western Qattara depression, Egypt: Contributions from the Museum of Paleontology, The University of Michigan, v. 32, no. 6, p. 71-90.

Zalmout, I. S., Antar, M. S. M., Hatab, E.-B. E., and Gingerich, P. D., 2011, Late Eocene vertebrate faunas from the Qattara Depression in the Western Desert of Egypt: Geological Society of America. Programs with Abstracts, v. 43, no. 5, p. 264.

Zalmout, I. S., and Gingerich, P. D., 2012, Late Eocene sea cows (Mammalia, Sirenia) from Wadi al Hitan in the Western Desert of Fayum, Egypt: University of Michigan Papers on Paleontology, v. 37, p. 1-158.

Zalmout, I. S., Mustafa, H. A., and Gingerich, P. D., 2000, Priabonian *Basilosaurus isis* (Cetacea) from the Wadi Esh-Shallala Formation: first marine mammal from the Eocene of Jordan: Journal of Vertebrate Paleontology, v. 20, no. 1, p. 201–204.

Zouhri, S., Gingerich, P. D., Elboudali, N., Bouzarar, W., and Omar, S., 2019a, Discovery of a new middle-upper Eocene vertebrate locality (Sabkha of Lebreij) in the Sahara Desert, southeast Morocco: International Symposium PalEurAfrica, p. 59.

Zouhri, S., Gingerich, P. D., Elboudali, N., Sebti, S., Noubhani, A., Rahali, M., and Meslouh, S., 2014, New marine mammal faunas (Cetacea and Sirenia) and sea level change in the Samlat Formation, Upper Eocene, near Ad-Dakhla in southwestern Morocco: Comptes Rendus Palevol, v. 13, no. 7, p. 599-610.

Zouhri, S., Gingerich, P. D., Khalloufi, B., Elboudali, N., Bouzarar, W., and Omar, S., 2019b, The middle-upper Eocene vertebrate record from Tarfaya-Laayoune Dakhla Atlantic Basin (Morocco): International Symposium PalEurAfrica, p. 60.
